# Métastases cutanées révélant un adénocarcinome bronchique

**DOI:** 10.11604/pamj.2016.24.2.9397

**Published:** 2016-05-03

**Authors:** Youssef Zemmez, Adil Zegmout, Jalal Hamama, Ahmed Bouhamidi, Mohammed El Amraoui, Jaouad El Azhari, Mohamed Boui

**Affiliations:** 1 Service de Dermatologie, Hôpital Militaire d'Instruction Mohammed V, Rabat, Maroc; 2 Service de Pneumologie, Hôpital Militaire d'Instruction Mohammed V, Rabat, Maroc; 3 Service de Chirurgie Plastique, Hôpital Militaire d'Instruction Mohammed V, Rabat, Maroc

**Keywords:** Métastases cutanées, adénocarcinome bronchique, biopsie-exérèse, Cutaneous metastases, bronchial adenocarcinoma, excisional biopsy

## Abstract

Nous rapportons le cas d'un cancer bronchique révélé par des nodules cutanés métastatiques du cuir chevelu. Ce mode de découverte assez fréquent est souvent associé à un mauvais pronostic. Cette observation souligne l'intérêt de rechercher un cancer primitif pulmonaire en cas de localisation secondaire cutanée.

## Abstract (en)

We report the case of bronchial carcinoma revealed by metastatic skin nodules on the scalp. This fairly common mode of discovery is often associated with poor prognosis. This study aims to underline the importance of directing the search for a primary lung cancer in cases of secondary skin involvement.

## Introduction

Les métastases cutanées peuvent accompagner près de 3 à 10% de tous les cancers confondus. Elles sont souvent décelées après le diagnostic de la néoplasie primitive. Cependant, leur découverte peut être synchrone, et celle de la métastase peut même être inaugurale et, dans ce cas, être appelée «précoce». L'identification de l'origine de la métastase s'avère parfois difficile. Le pronostic de la maladie au stade métastatique est habituellement sombre. Nous rapportons le cas d'un adénocarcinome bronchique révélé par des nodules cutanés métastatiques du cuir chevelu.

## Patient et observation

**Figure 1:**
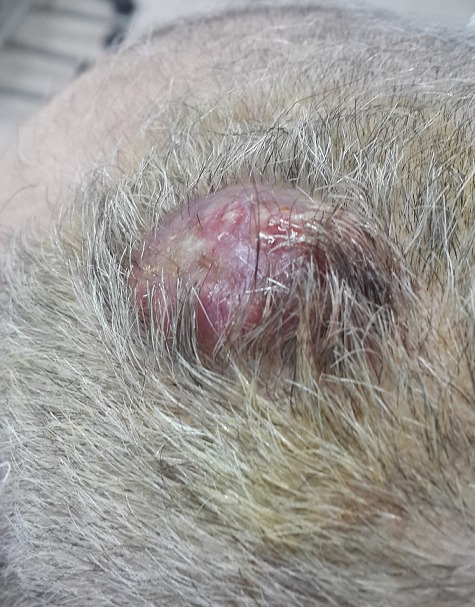
Nodules cutanés du cuir chevelu

**Figure 2:**
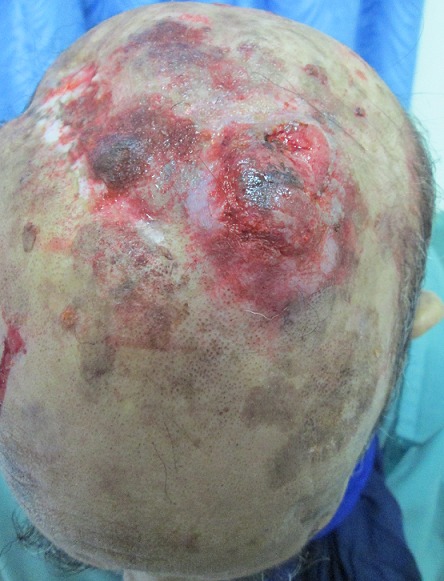
Biopsie exérèse des lésions du cuir chevelu

**Figure 3:**
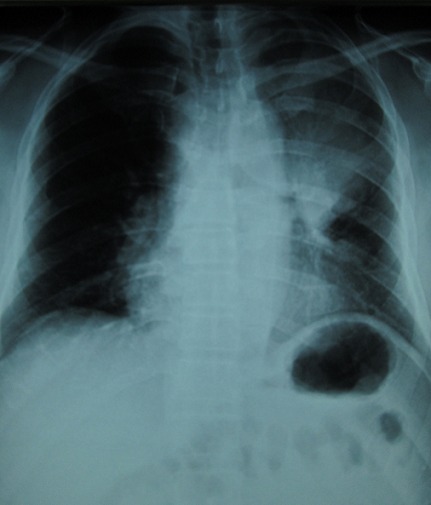
Radiographie thoracique de face: opacité Para-hilaire gauche

**Figure 4:**
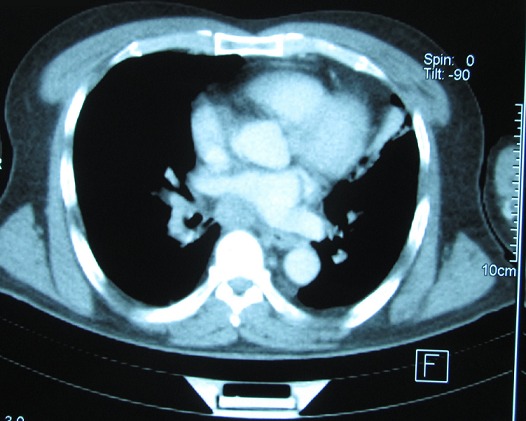
TDM thoracique: processus tumoral para-hilaire gauche

Monsieur A.M âgéde 60 ans, tabagique chronique à raison de 30 paquets années, a consulté en dermatologie pour des lésions indolores du cuir chevelu de taille rapidement croissante. A l'examen clinique, il s'agissait de 3 nodules cutanés de type angiomateux de 2 cm de diamètre avec un caractère ferme et indolore siégeant au niveau pariétal et occipital (Figure 1). Le tout évoluant dans un contexte d'apyrexie et d'altération de l’état général, sans tendance à la régression spontanée des lésions. Le reste de l'examen était normal en particulier les aires ganglionnaires. Le patient a subit une biopsie exerese (Figure 2) d'un nodule siegeant au niveau pariétal, dont l'histologie était en faveur d'une localisation secondaire d'un adénocarcinome d'origine pulmonaire (TTF1 positif). La radiographie thoracique de face montrait une opacité para-hilaire gauche, la TDM thoracique objectivait un processus tissulaire nécrotique para-hilaire gauche (Figure 3, Figure 4). La fibroscopie bronchique objectivait un bourgeon tumoral dont l’étude anatomopathologique était en faveur d'un adénocarcinome bronchique. Le bilan d'extension (scintigraphie osseuse et IRM cérébrale) n'a pas montré d'autres localisations secondaires. La décision thérapeutique était de commencer une poly-chimiothérapie.

## Discussion

Les métastases cutanées ne sont pas rares puisque 3 à 10% des cancers en sont à l'origine [1, 2]. La majorité des métastases cutanées trouvent leur origine au niveau d'un mélanome cutané ou d'un carcinome cutanéo-muqueux ou neuroendocrine. En principe, tout cancer peut métastaser dans la peau, le plus souvent après la découverte dela néoplasie primitive. Parfois cependant, il y a simultanéité entre la survenue d'une métastase cutanée et la révélation de sa néoplasie primitive. Enfin, la métastase peut apparaître isolée, alors que le cancer primitif n'a pas encore été détecté ou a régressé complètement [3]. Chez l'homme, cette dernière situation doit orienter prioritairement l'exploration vers un cancer pulmonaire ou rénal. Chez la femme, les reins et les ovaires devraient être ciblés [4]. Des métastases très tardives, survenant plus de 10 ans après l’éradication de la néoplasie primitive sont possibles, en particulier en cas de mélanomes et de divers cancers du sein, du rein, de la vessie, du côlon, de la prostate, de l'ovaire et du larynx. Il existe plusieurs voies distinctes de dissémination métastatique jusqu’à la peau. L'extension directe de la néoplasie par contiguïté est possible, et elle est même fréquente en cas de cancer mammaire. La dissémination par voie lymphatique ou hématogène est classiquement reconnue. La dissémination lors de l'intervention chirurgicale sur le néoplasme primitif est une autre éventualité. Une voie distincte, apparemment typique du mélanome, consiste en la migration des cellules néoplasiques le long de la face externe des vaisseaux [3, 5]. Les présentations cliniques des métastases sont variées. Bien souvent, il y a une relation de proximité entre le cancer primitif et les localisations métastatiques cutanées. Les nodules métastatiques sont en général peu nombreux et ils peuvent adopter un regroupement régional variable selon la nature du cancer primitif. Ils sont fermes et habituellement non douloureux. Pouvant atteindre quelques centimètres de diamètre, ils apparaissent subitement. Leur croissance est habituellement rapide avant de se stabiliser dans leur expansion, sans cependant avoir tendance à la régression spontanée. Parfois, les métastases deviennent bulleuses ou érodées. D'autres ont un aspect inflammatoire érysipéloïde [6], voire scléreux ou en cuirasse. Les métastases des néoplasies pulmonaires se localisent surtout sur le thorax [7]. Celles du côlon et du rectum se situent le plus souvent au niveau de la paroi abdominale, en particulier au niveau d'une cicatrice, et dans la région périnéale [7]. Le nodule de soeur Mary-Joseph localisé au niveau de l'ombilic a souvent pour origine une néoplasie de l'estomac, du gros intestin, de l'ovaire ou du pancréas [8, 9].

## Conclusion

Bien que toute néoplasie maligne puisse métastaser dans la peau, seul un petit groupe de cancers est régulièrement impliqué. L'aspect clinique, la topographie des lésions et le sexe du malade sont importants à considérer. L'examen histologique et son complément immunohistochimique apportent bien souvent la clé du diagnostic. Il reste cependant de rares cas où l'identification de la métastase et de son origine restent un défi non satisfait.
